# The Preventive Effect of Cardiac Sympathetic Denervation Induced by 6-OHDA on Myocardial Ischemia–Reperfusion Injury: The Changes of lncRNA/circRNAs-miRNA-mRNA Network of the Upper Thoracic Spinal Cord in Rats

**DOI:** 10.1155/2021/2492286

**Published:** 2021-11-29

**Authors:** Zhixiao Li, Yujuan Li, Zhigang He, Zhen Li, Weiguo Xu, HongBing Xiang

**Affiliations:** ^1^Department of Anesthesiology and Pain Medicine, Tongji Hospital of Tongji Medical College, Huazhong University of Science and Technology, Wuhan, 430030 Hubei, China; ^2^Department of Orthopedics, Tongji Hospital of Tongji Medical College, Huazhong University of Science and Technology, Wuhan 430030, China

## Abstract

In this study, we investigated whether chemical 6-hydroxydopamine (6-OHDA) stimuli caused cardiac sympathetic denervation (SD), and we analyzed gene expression profiles to determine the changes in the lncRNA/circRNAs-miRNA-mRNA network in the affected spinal cord segments to identify putative target genes and molecular pathways in rats with myocardial ischemia–reperfusion injury (MIRI). Our results showed that cardiac sympathetic denervation induced by 6-OHDA alleviated MIRI. Compared with the ischemia reperfusion (IR, MIRI model) group, there were 148 upregulated and 51 downregulated mRNAs, 165 upregulated and 168 downregulated lncRNAs, 70 upregulated and 52 downregulated circRNAs, and 12 upregulated and 11 downregulated miRNAs in the upper thoracic spinal cord of the SD-IR group. Furthermore, we found that the differential genes related to cellular components were mainly enriched in extracellular and cortical cytoskeleton, and molecular functions were mainly enriched in chemokine activity. Pathway analysis showed that the differentially expressed genes were mainly related to the interaction of cytokines and cytokine receptors, sodium ion reabsorption, cysteine and methionine metabolism, mucoglycan biosynthesis, cGMP-PKG signaling pathway, and MAPK signaling pathway. In conclusion, the lncRNA/circRNAs-miRNA-mRNA networks in the upper thoracic spinal cord play an important role in the preventive effect of cardiac sympathetic denervation induced by 6-OHDA on MIRI, which offers new insights into the pathogenesis of MIRI and provides new targets for MIRI.

## 1. Introduction

Myocardial ischemia/reperfusion injury (MIRI) accounts for a large proportion of the total incidence of heart diseases, and it seriously affects human quality of life [1–3]. Previous studies have reported the cellular and molecular mechanisms of neural–cardiac interactions [4, 5] during pathological remodeling after MIRI [6]. However, to date, no effective methods have been found to prevent MIRI.

Cardiac nerves, comprising both the sensory nerves and the autonomic nerves, transmit the information from the heart to the spinal cord and brain, which then results in an appropriate sympathetic neural outflow [7]. The role of sympathetic activity in the development of cardiac electrical activity has been well known for decades [8, 9]. Neural regulation is involved in an imbalance between the sympathetic and parasympathetic nervous systems within the ischemic myocardial tissues. Experimental studies have shown that cardiac innervation abnormality is an important cause of the sympathetic nervous system overactivity. Several studies have reported the vital role of the sympathetic nerves in MIRI progression, and sympathetic nerves have been shown to infiltrate the myocardial microenvironment thereby accelerating cardiac injury [10, 11]. In this study, we examined the preventive effect of cardiac sympathetic denervation induced by 6-OHDA, a catecholamine-specific toxin [12], on MIRI rats.

Myocardial ischemia/reperfusion injury is a complex process, and further understanding of the related biological processes and their regulation is necessary [13, 14]. Several lines of evidence have revealed a close correspondence between the spinal cord and cardiovascular system [15–17]; indeed, the spinal cord and cardiovascular system develop in concert and are functionally interconnected in heart disease. It is an accepted fact that noncoding RNAs (ncRNAs) of the spinal cord are vital components of the regulation and cross-talk among MIRI-related signaling pathways [16]. In recent years, a strong consensus has been reached that ncRNAs, including long noncoding RNAs (lncRNAs) and circular RNAs (circRNAs), play an important role in many cellular processes and occurrence of diseases [18–23]. However, the underlying mechanisms based on the function of lncRNAs, circRNAs, and mRNAs in the spinal cord following MIRI remain unclear. Thus, it is necessary to analyze the lncRNAs and circRNAs comprehensively and explore the role of the lncRNA/circRNAs-miRNA-mRNA network in MIRI.

In this study, we first investigated whether cardiac sympathetic denervation induced by 6-OHDA alleviates MIRI. Next, we performed high-throughput sequencing on the spinal cord tissues for the first time to describe and analyze the expression profiles of ncRNAs, including lncRNA and circRNA. Furthermore, we performed Gene Ontology (GO) and Kyoto Encyclopedia of Genes and Genomes (KEGG) analysis of differentially expressed lncRNAs and circRNAs. We also constructed lncRNA/circRNA-miRNA-mRNA networks to further explore their underlying mechanism and possible relationships in the preventive effect of cardiac sympathetic denervation induced by 6-OHDA on MIRI.

## 2. Materials and Methods

### 2.1. Animals

Adult male Sprague–Dawley rats (weighing 250–300 g) were used, and two animals were placed in each cage. The animals had free access to food and water and were housed in a light- and temperature-controlled room. The experiment started following the approval of the Institutional Animal Care and Use Committee in Tongji Hospital, Huazhong University of Science and Technology (approval No. TJ-A0804).

### 2.2. Groups and Chemical Sympathetic Denervation

The rats were randomly assigned to the following two groups: MIRI model (IR) group and sympathetic denervation (SD) + IR (SD-IR) group (n = 9 for each group). In the SD-IR group, intraperitoneal (i.p.) injections of 50 mg/kg 6-hydroxydopamine (6-OHDA, Sigma), containing 0.1% ascorbic acid in the saline solution of 6-OHDA, were administered for 3 consecutive days [1, 24, 25], whereas MIRI rats received i.p. injections of the same volume of saline. One day after the last injection, rats (n = 6 for each group) were deeply anesthetized, and left ventricular tissues were harvested for further laboratory study, whereas other rats received the establishment of MIRI model.

### 2.3. Establishment of the MIRI Model

The MIRI model was modified from a previous study [3, 15, 16, 26, 27]. In brief, after routine disinfection, anesthesia, and tracheal intubation, surgical thoracotomy was conducted. In both groups (n = 9 for each group), the left anterior descending coronary artery (LAD) was ligated 2 mm below the left atrial appendage for 30 minutes and then reperfused for 2 hours. The core temperature was maintained throughout the protocol. The rats were monitored to confirm ischemic ST segment elevation during LAD occlusion by an electrocardiogram. Serum troponin cTnl of the two groups was measured 2 hours after reperfusion as an index of myocardial necrosis. Hearts were harvested for hematoxylin and eosin (H&E) staining and 2,3,5-triphenyl tetrazolium chloride (TTC) staining.

### 2.4. Determination of Norepinephrine Content

The norepinephrine (NE) content of myocardial tissue from the left ventricle was measured using a high-performance liquid chromatography (HPLC) method [28–31]. In brief, cardiac tissue of the left ventricle was weighed (about 100 mg) and homogenized in 500 *μ*L of precooled methanol/water (V:V = 2/1). The homogenate mixture was sonicated (12000 rpm, 10 minutes, 4°C) and the supernatant was collected, while the remaining pellets were repeatedly treated twice. Three supernatants were combined and dried, and then redissolved in 100 *μ*L formic acid solution (0.1%). Next, the following chemicals were added in turn to the supernatant (10 *μ*L): NEM solution (2.5 mM, 80 *μ*L), tBBT solution (1 M in DMSO, 10 *μ*L), borate buffer (0.2 M, pH 8.8, 700 *μ*L), 5-AIQC solution (200 *μ*L), formic acid (10 *μ*L). The solution was sonicated (12000 rpm, 10 minutes, 4°C) and the supernatant was filtered by a 0.22 *μ*m membrane filter before HPLC analysis. HPLC analyses were conducted on the 1290-6470 UPLC-MS/MS system (Agilent, USA). Data preprocessing was performed using Mass Hunter Workstation software (Agilent, Version B.08.00).

### 2.5. Myocardial Tissue Staining

Myocardial tissue staining was performed as described previously [15]. In brief, each myocardial tissue sample was cut transversely. Hematoxylin and eosin (H&E) staining was used to observe myocardial pathology. After deparaffinization, 4-*μ*m-thick sections were immersed in hematoxylin (cat. no. H9627; Sigma-Aldrich; Merck KGaA) for 5–7 min at room temperature, differentiated in 1% acid alcohol for 2–5 seconds, and stained with 0.5% eosin (cat. no. 71014544; Sinopharm Chemical Reagent Co., Ltd.) for 2 minutes at room temperature.

After rinsing with distilled water for 30 seconds, the sections were dehydrated with graded alcohols and cleared in xylene. Infarct size was assessed by TTC staining 2 hours after reperfusion. After surgery, the hearts were removed and frozen for 20 minutes at −20°C, and then transversally cut into sections with a thickness of 1–2 mm. The tissue sections were incubated for 10 minutes in 2% TTC in dark conditions at 37°C and then fixed overnight in 10% formaldehyde at 4°C. The infarct area was white, while the normal tissues were red.

Infarct size and area at risk (AAR) in TTC-stained cardiac sections of the left ventricle were determined as previously described [32]. Briefly, Pale regions were regarded as the areas of necrosis (AON). AON and AAR were calculated as the average percent area per slice from both sides of each section. Then, they were normalized to slice weight as follows: weight of total AAR = (weight of slice 1 × % AAR of slice 1) + (weight of slice 2 × % AAR of slice 2) + (weight of slice 3 × % AAR of slice 3) + (weight of slice 4 × % AAR of slice4) + (weight of slice 5 × % AAR of slice 5). AON weight was calculated in the same manner.

Finally, infarct size was expressed as the percentage of the weight of AON to the weight of AAR [33]. If the AAR was >90%, this animal was excluded.

### 2.6. RNA Sequencing for lncRNA-circRNA-mRNA

Two hours after reperfusion, the animals were quickly sacrificed to limit their suffering. The upper thoracic (T1–T4) spinal cord segments were immediately cut and frozen with liquid nitrogen and sent to Oebiotech Corporation (Shanghai, China) for RNA sequencing. The extraction of total RNA from T1–T4 spinal tissues was conducted by using the mirVana™ PARIS™ Kit (Ambion-1556, USA) in accordance with the user manual. We assessed total RNA integrity using Agilent Bioanalyzer 2100 (Agilent Technologies). The spinal samples from the two groups were selected for microarray analysis. We used the Affymetrix® GeneChip® Whole Transcript Expression Arrays to analyze lncRNA and mRNA expression profiles, and we applied Agilent circRNA Microarray 8x60K to analyze circRNA expression profiles in the T1–T4 spinal cord segments [34]. Microarray data were obtained and analyzed by Oebiotech Corporation (Shanghai, China). The RNA-sequencing results were used to prioritize the heart-related spinal genes.

### 2.7. Identifying Differentially Expressed Genes and Gene Ontology (GO) Analysis

The differentially expressed mRNAs, lncRNAs, and circRNAs from the RNA-seq data were identified by using the edgeR algorithm. The mRNAs, lncRNAs, and circRNAs were deemed differentially expressed if they showed a false-discovery rate (FDR) < 5% and fold change (FC) > 2. The molecular functions, cellular components, and biological processes of differentially expressed lncRNAs and circRNAs were described by using GO analysis (http://www.geneontology.org).

### 2.8. Kyoto Encyclopedia of Genes and Genomes (KEGG) Pathway Analysis

The gene set scores were calculated by using the FAIME algorithm based on the rank-weighted gene expression levels of individual samples (from the T1–T4 segments of the spinal cord), which converts each sample's transcriptomic profile to molecular mechanisms. KEGG analysis was applied to determine the biologic pathway roles of these differentially expressed lncRNAs and circRNAs based on the latest KEGG data (http://www.genome.jp/kegg/). Student's *t* test was used to identify the differentially expressed KEGG pathways between IR and SD-IR samples. The KEGG pathways with adjusted p < 0.05 by Benjamini–Hochberg procedure were considered differentially expressed.

### 2.9. RT-qPCR Analysis for the Upper Thoracic Spinal Cord

The total RNA extracted from the T1–T4 segments of the spinal cord using the TRIzol® reagent (Invitrogen; Thermo Fisher Scientific, Inc.) in line with the manufacturer's instructions was used for the generation of cDNA. The primers were designed using the Primer Express 3.0 software (Applied Biosystems; Thermo Fisher Scientific, Inc.); the specific forward and reverse primer sequences are listed in Table 1. RNA samples were quantified using a spectrophotometer (BioPhotometer; Eppendorf AG) and then synthesized to cDNA by reverse transcription using the PrimeScript™ RT reagent kit (Takara Bio, Inc.). The temperature protocol was conducted using the following protocol: 15 minutes at 37°C, 5 seconds at 85°C, and held at 4°C. cDNA was quantitated via RT-qPCR using a TB green® Premix Ex Taq (cat. no. RR420A; Takara Bio, Inc.). The thermocycling conditions for PCR were as follows: Initial denaturation for 30 seconds at 95°C, followed by 40 cycles of 15 seconds at 95°C, 15 seconds at 60°C, and 45 seconds at 72°C. The threshold cycle (Cq) was used to estimate the amount of target mRNA. The comparative Cq method with a formula for relative FC (2^-*ΔΔ*Cq^) was used to quantify the amplified transcripts. The relative gene expression levels were determined via normalization to GAPDH. Experiments were evaluated in triplicate and repeated ≥3 times.

### 2.10. Construction of lncRNA/circRNA-miRNA-mRNA Network

The TargetScan (Release 7.2) and Miranda (version 0.10.80) software were used to predict the relationship among lncRNA/circRNA, miRNAs, and mRNAs through base pairing. These predicted results were integrated to build the potential lncRNA/circRNA-miRNA-mRNA network. The Cytoscape software (version 3.7.2) was used to visualize the above data so as to explore the role of lncRNA/circRNA-miRNA-mRNA ceRNA network in the pathogenesis of MIRI after cardiac sympathetic denervation.

### 2.11. Computational Prediction of Protein–Protein Interaction (PPI) Analysis

The STRING database (ver. 10.5; https://string-db.org/) is an online database tool for searching known or predicted information on PPIs. The minimum PPI interaction score was set at 0.900 (highest confidence), and the wide disconnected node in the network was observed to obtain a complex PPI network of differentially expressed mRNAs. The Cytoscape software (version 3.7.2) was used to visualize the PPI network, and Cytohubba (a plug-in of Cytoscape) was used to identify the most relevant nodes by setting the degree. The PPI analysis was limited to an interaction threshold of 0.4 (medium confidence).

### 2.12. Statistical Analysis

Data were presented as the mean ± SEM and were analyzed using GraphPad Prism software v5.0 (GraphPad Software, Inc.). Based on Gene Ontology Biological Process (GOBP) definition, Fisher's exact test was applied to determine whether the proportion of differentially expressed genes in a given GOBP gene set was significantly enhanced. RT-qPCR parameters were analyzed using the unpaired *t* test for repeated measures, with *P* value less than 0.05 was considered statistically significant.

## 3. Results

### 3.1. Chemical Sympathectomy-Induced Cardiac Alterations

**Figure 1:**
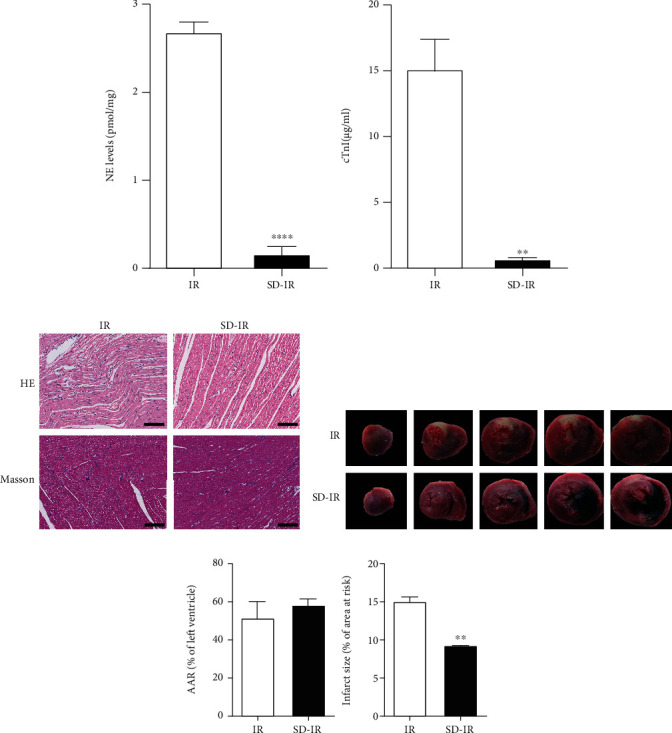
Chemical sympathetic denervation attenuates myocardial ischemia–reperfusion injury. (a) Serum NE and (b) serum cTnI concentrations in rats with IR or SD-IR. (c) Representative images of hematoxylin and eosin staining and Masson trichrome staining of rat hearts 24 h after IR injury. Scale bar = 100 *μ*m. (d) Representative photographs of TTC-Evan blue staining in hearts subjected to IR and SD-IR surgery. (e) Quantification of AAR and infarct area vs. AAR in rat hearts in IR group and SD-IR group. Data are expressed as mean ± SEM. ∗∗*p* < 0.01, ∗∗∗∗*p* < 0.0001 vs. IR group. NE: norepinephrine; IR: ischemia reperfusion; AAR: area at risk.

The level of NE in the cardiac tissues of the SD-IR group (n = 6, 0.1650 ± 0.1057 ng/mg) was significantly lower compared with that in the IR group (n = 6, 2.687 ± 0.1349 ng/mg) (Figure 1(a)).

### 3.2. Characteristics of Myocardial Ischemic Tissue

In the two groups, we observed the development of ST-segment elevation and QRS complex changes on an electrocardiogram; moreover, there was a cyanotic change in the myocardium of the occluded area 30 minutes after cardiac ischemia. Serum cardiac troponin cTnI (0.73 ± 0.26 *μ*g/L) in the SD-IR group was significantly lower than that in the IR group (15.14 ± 2.44 *μ*g/L) 2 hours after reperfusion (Figure 1(b)). These results verified the successful occlusion of the LAD.

In the IR group, a structural disorder of the cardiac tissue was observed, with different degrees of vacuolar degeneration and necrosis, as well as loose stroma (Figure 1(c)). Moreover, the number of cardiomyocyte fibers was markedly increased after IR. In the SD-IR group, myocardium showed a better architecture, and the myocardial fibers and myocardial cells were relatively intact and arranged in an orderly manner (Figure 1(c)).

The results from TTC staining clearly exhibited a reduced myocardial infarction—indicated by the pale color region in the transverse section of heart—in the IR group (Figure 1(d)). To ensure that the difference in the infarct size was not caused by different myocardium injuries, we measured the area at risk (AAR) and found no difference between the two groups (Figure 1(e)). TTC staining showed a significant reduction in infarct size in the SD-IR group (9.26 ± 0.16%, n = 6) compared with the IR group (15.05 ± 0.70%, n = 6) (Figure 1(e)).

### 3.3. Differential Expression of lncRNAs, circRNAs, miRNA, and mRNAs

**Figure 2:**
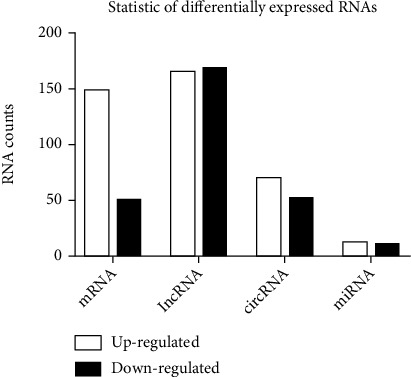
Differentially expressed RNAs. Through high-throughput RNA sequencing, we found that 148 mRNAs were upregulated and 51 mRNAs were downregulated; 165 lncRNAs were upregulated and 168 lncRNAs were downregulated; 70 circRNAs were upregulated and 52 circRNAs downregulated; and 12 miRNAs were upregulated and 11 miRNAs were downregulated.

To fully understand the role of cardiac sympathetic denervation in myocardial ischemia/reperfusion injury, we simultaneously analyzed the profiles of differential expression of lncRNAs, circRNAs, miRNAs, and mRNAs through microarray analysis. Significant difference was defined as fold change ≥2 and p < 0.05. In this study, the SD-IR group identified 333 lncRNAs, 122 circRNAs, 23 miRNAs, and 199 mRNAs with significant differential expression (Figure 2). Through high-throughput RNA sequencing, we found that 165 lncRNAs were upregulated and 168 lncRNAs were downregulated; 70 circRNAs were upregulated and 52 circRNAs were downregulated; 12 miRNAs were upregulated and 11 miRNAs were downregulated; and 148 mRNAs were upregulated and 51 mRNAs were downregulated (Figure 2).

**Figure 3:**
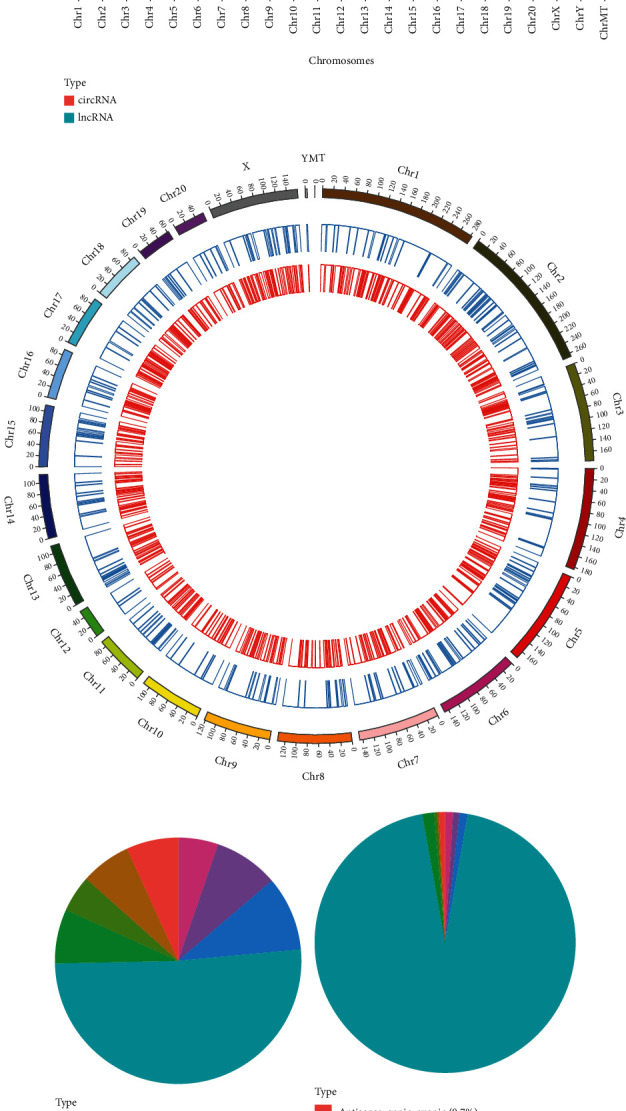
Comparison of the characteristics of lncRNAs, circRNAs, and mRNAs expression profiles in IR group and SD-IR group. (a) The distribution of lncRNAs and circRNAs on chromosomes. (b) LncRNAs and mRNAs on chromosomes. (c, d) Classification of lncRNAs and circRNAs.

A total of 23711 lncRNAs and 12007 circRNAs (Figures 3(a) and 3(b)) were identified in all chromosomes. Among lncRNAs, most (51.1%) were sense_genic_exonic lncRNA, followed by sense_genic_intronic lncRNAs (9.8%), sense_intergenic_downstream lncRNAs (8.5%), sense_intergenic_upstream lncRNAs (5.3%), antisense_genic_exonic lncRNA (6.8%), antisense_genic_intronic lncRNAs (6.6%), antisense_intergenic_downstream lncRNAs (4.8%), and antisense_intergenic_upstream lncRNAs (7.1%) (Figure 3(c)). In circRNA, the vast majority (94.4%) were sense_genic_exonic circRNA, followed by sense_genic_intronic circRNAs (1%), sense_intergenic_downstream circRNAs (0.8%), sense_intergenic_upstream circRNAs (1%), antisense_genic_exonic circRNA (0.7%), antisense_genic_intronic circRNAs (0.2%), antisense_intergenic_downstream circRNAs (0.4%), and antisense_intergenic_upstream circRNAs (1.5%) (Figure 3(d)).

### 3.4. Differential Expression Patterns of mRNAs, lncRNAs, and circRNAs in MIRI

**Figure 4:**
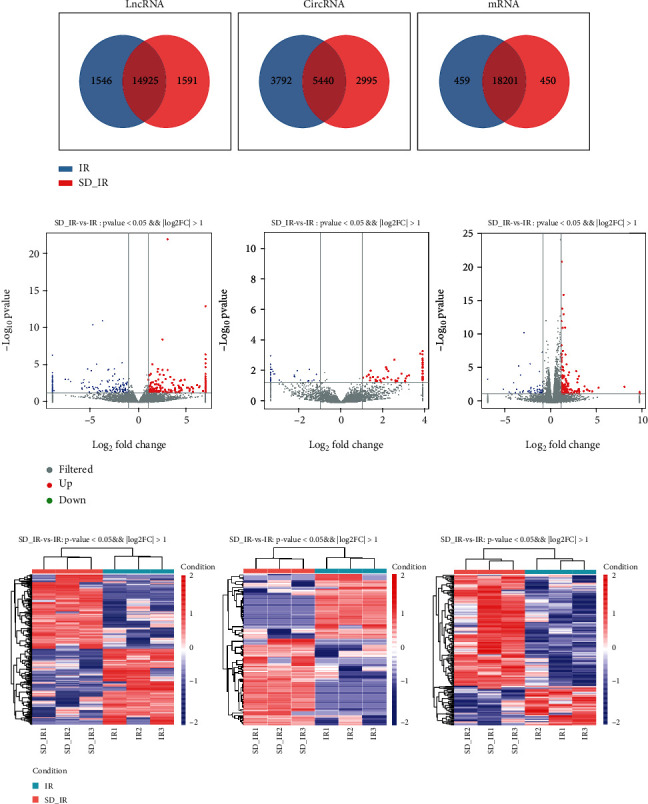
Differential expression patterns of lncRNAs, circRNAs, and mRNAs. (a) The specific lncRNAs, circRNAs, and mRNAs shared between IR group and SD-IR group. (b) The volcano plot of lncRNAs, circRNAs, and mRNAs expression. Red color is indicative of upregulated and blue color is indicative of downregulated genes, where p < 0.05 and |FC| > 2 are considered statistically significant; grey color is indicative of nonsignificantly different genes. (c) The hierarchical heat map shows the deregulated lncRNAs, circRNAs, and mRNAs in the T1–T4 spinal cord segments between IR group and SD-IR group; up- and downregulated genes are colored in red and green, respectively (p < 0.05 and log_2_|FC| > 1).

The expression patterns of mRNAs, lncRNAs, and circRNAs in the T1–T4 spinal cord 2 hours after MIRI were examined using microarray. From the volcano map and hierarchical clustering analysis results between the SD-IR group and the IR group, a landscape of the expression characteristics of the mRNAs, lncRNAs, and circRNAs was obtained (Figure 4(a)). The volcano plots demonstrated that large numbers of mRNAs, lncRNAs, and circRNAs were differentially expressed between the two groups (Figure 4(b)). Furthermore, these differential alterations of mRNA, lncRNA, and circRNA expression in the T1–T4 spinal cord were associated with cardiac sympathetic denervation. The hierarchical heat map showed the deregulated mRNAs, lncRNAs, and circRNAs in the T1–T4 spinal cord segments between the SD-IR group and the IR group (Figure 4(c)); up- and downregulated genes are colored in red and green, respectively (p < 0.05 and log2|FC| > 1).

### 3.5. Analysis of mRNAs, lncRNAs, circRNAs, and miRNAs Changes in the Spinal Cord after Cardiac Sympathetic Denervation

Among the differentially expressed mRNAs, there were 199 genes exhibiting fold change (FC) higher than 1. The number of downregulated mRNAs was 51, whereas the number of upregulated mRNAs was 148. The most upregulated mRNAs were Fxyd2, Tyrp1, Il31ra, RT1-CE4, Scn11a, MGC108823, Tnc, Irf8, and MGC105567. The most downregulated mRNAs were LOC100911256, Cyp4b1, LOC103689986, LOC103693165, Sh2d4b, LOC100910308, Nfs1, Prex2, LOC103691806, and Hif3a. The detailed information regarding the differentially expressed mRNAs is listed in Tables 2 and 3.

The results showed that 333 lncRNAs, including 165 upregulated and 168 downregulated lncRNAs, were significantly altered in the SD-IR group compared with the IR group. The most upregulated lncRNAs were NONRATT003225.2, TCONS_00000042, NONRATT013473.2, NONRATT011603.2, NONRATT018299.2, NONRATT001917.2, NONRATT017719.2, NONRATT004831.2, TCONS_00008547, and NONRATT025548.2. The most downregulated lncRNAs were NONRATT003165.2, NONRATT006541.2, NONRATT031746.1, TCONS_00013588, TCONS_00009293, NONRATT007713.2, NONRATT016595.2, NONRATT011191.2, NONRATT025664.2, and NONRATT010240.2. Additional information regarding the differentially expressed lncRNAs is presented in Table 4.

Compared with the IR group, the SD-IR group showed 70 upregulated and 52 downregulated circRNAs. The most upregulated circRNAs were circRNA_00336, circRNA_00446, circRNA_00547, circRNA_01552, circRNA_01962, circRNA_01988, circRNA_02159, circRNA_02217, circRNA_03207, and circRNA_03748. The most downregulated circRNAs were circRNA_02339, circRNA_02911, circRNA_03499, circRNA_03573, circRNA_04050, circRNA_04274, circRNA_04457, circRNA_04622, circRNA_06863, and circRNA_07224. Additional information regarding the differentially expressed circRNAs is presented in Tables 5 and 6.

Among the differentially expressed miRNAs, there were 12 upregulated and 11 downregulated miRNAs in the SD-IR group compared with the IR group. The upregulated miRNAs were rno-miR-293-5p, rno-miR-183-5p, rno-miR-96-5p, rno-miR-493-5p, novel248_mature, rno-miR-363-3p, novel21_star, rno-miR-146a-3p, novel98_mature, rno-miR-3553, novel438_mature, and novel174_mature>novel176_mature>novel705_mature. The downregulated miRNAs were novel586_mature, novel88_mature, novel342_mature, rno-miR-206-3p, novel190_mature, rno-miR-1-3p, novel655_mature, novel62_mature, rno-miR-7a-2-3p, novel252_mature, and novel275_mature>novel301_mature. Additional information regarding the differentially expressed miRNAs is shown in Table 7.

### 3.6. GO and KEGG Analysis of Differentially Expressed mRNAs, lncRNAs, circRNAs, and miRNAs

To investigate the spinal molecular mechanisms of cardiac sympathetic denervation on MIRI, we performed GO and KEGG pathway analyses of the differentially expressed mRNAs (DEM), lncRNAs (DEL), circRNAs (DEC), and miRNAs in SD-MIRI vs. IR group (Tables 8 and 9).

**Figure 5:**
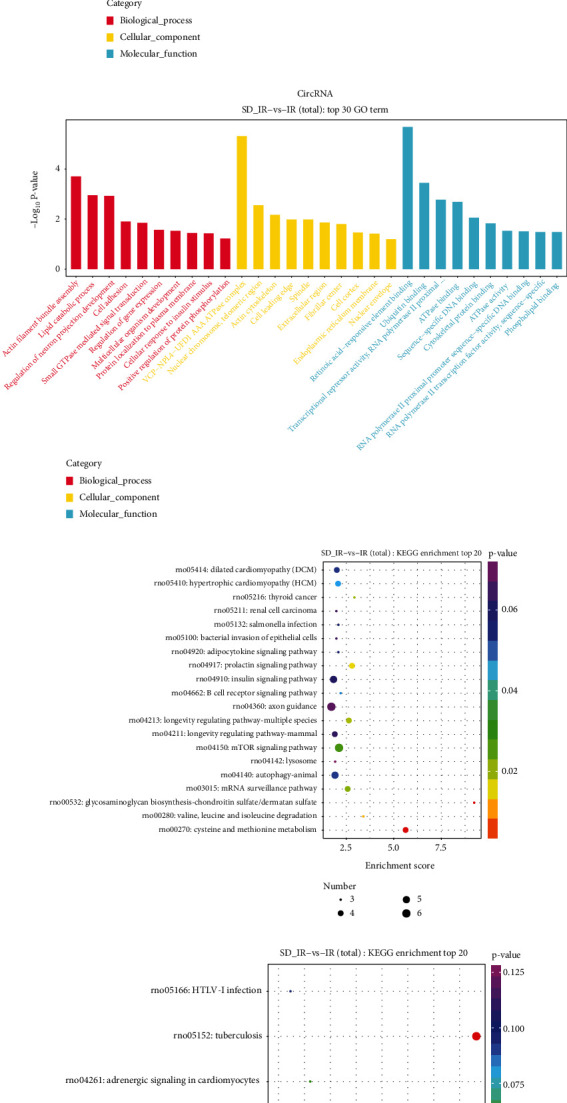
Functional analysis of the differentially expressed lncRNAs and circRNAs between IR group and SD-IR group. (a) The top 30 Gene Ontology terms of differentially expressed lncRNAs. Green color is related to biological processes; blue color is related to cellular components; and red color is related to molecular functions. (b) The top 30 Gene Ontology terms of differentially expressed circRNAs. Richly factor refers to the ratio of the number of differentially expressed genes in the KEGG pathway accounting for the total number of genes that are related to this pathway. The larger the richly factor, the higher the degree of enrichment; the size of the bubble indicates the number of genes, which is qualified by Q-value. (c) The top 20 KEGG pathway enrichment analysis of differentially expressed lncRNAs. (d) The top six KEGG pathway enrichment analysis of differentially expressed circRNAs.

Based on GO analysis, DEL focusing on cell components were related to the Golgi cisterna, membrane, axon, cytoplasm, and cytoplasmic microtubules, while those focusing on molecular function (MF) were related to translation initiation factor activity, protein tyrosine kinase binding, myosin binding, and SNAP receptor activity (Figure 5(a)). The GO function prediction showed that DEC focusing on cell components were related to the VCP-NPL4-UFD1 AAA ATPase complex, nuclear chromosome telomeric region, and actin cytoskeleton, whereas those focusing on molecular functions (MF) were related to retinoic acid-responsive element binding, ubiquitin binding, ATPase activity, and sequence-specific DNA activity (Figure 5(b)).

KEGG analysis revealed the potential mechanism of DEL and DEC in the SD-IR group (Figures 5(c) and 5(d)). Namely, DEL are involved in the regulation of dilated cardiomyopathy (DCM), hypertrophic cardiomyopathy (HCM), insulin signaling pathway, and mTOR signaling pathway (Figure 5(c)). The parent gene of DEC might take part in MAPK signaling pathway, cGMP-PKG signaling pathway, protein processing in endoplasmic reticulum, and adrenergic signaling in cardiomyocytes (Figure 5(d)).

### 3.7. Verification of Differentially Dysregulated mRNAs and lncRNAs

**Figure 6:**
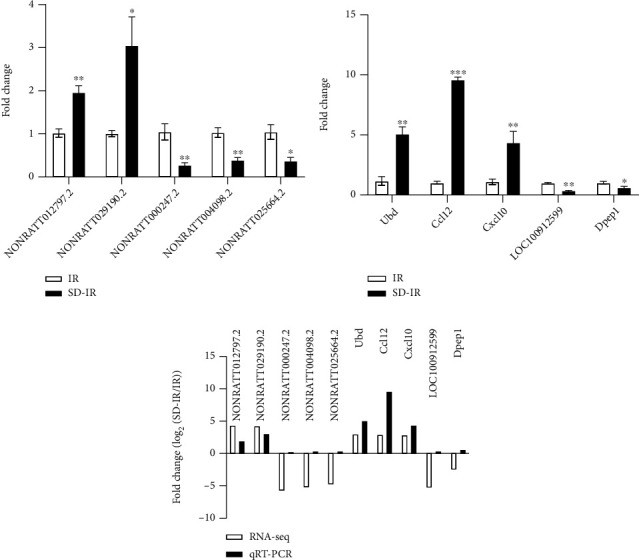
Validation of lncRNAs and mRNAs by RT-qPCR in the T1–T4 spinal cord IR group and SD-IR group. (a) The expression levels of lncRNA NONRATT012797.2 and NONRATT029190.2 were significantly upregulated in SD-IR group, whereas the expression levels of lncRNA NONRATT000247.2, NONRATT004098.2, and NONRATT025664.2 were significantly downregulated in SD-IR group. (b) The expression levels of mRNA Ubd, Ccl12, and Cxcl10 were significantly upregulated in SD-IR group, whereas the expression levels of mRNA LOC100912599 and Dpep1 were significantly downregulated in SD-IR group. (c) The expression levels of five lncRNAs and five mRNAs. Two upregulated lncRNAs, three downregulated lncRNAs, three upregulated mRNAs, and two downregulated mRNAs were validated by RT-qPCR. Data are expressed as mean ± SEM. ∗*P* < 0.05, ∗∗p < 0.01, ∗∗∗p < 0.001 vs. IR group.

We focused on the differentially dysregulated mRNAs and lncRNAs with more significant changes. Compared with the IR group, the lncRNAs selected in the SD-IR group, including NONRATT012797.2 and NONRATT029190.2, were significantly overexpressed and consistent with the RNA-sequencing results (Figures 6(a) and 6(c)). The lncRNAs selected in the SD-IR group, including NONRATT000247.2, NONRATT004098.2 and NONRATT025664.2, were significantly downregulated compared with the control group and were consistent with the RNA-sequencing results (Figures 6(a) and 6(c)).

For further research, we selected three upregulated mRNAs (Ubd, Ccl12, Cxcl10) and two downregulated mRNAs (LOC100912599, Dpep1) (Figures 6(b) and 6(c)) in the SD-IR group for RT-qPCR verification, *p* value <0.05, fold change ≥2. The primers of mRNAs and lncRNAs are listed in Table 1. Therefore, these results proved the accuracy of the microarray results.

### 3.8. Construction of the mRNA-miRNA-lncRNA/circRNA Network

CircRNA participates in the regulation of biological processes in different ways. It is well known that circRNA contains multiple binding sites of miRNA and is also regulated by miRNA. Analysis of circRNA-miRNA interaction may clarify the function and mechanism of circRNA.

**Figure 7:**
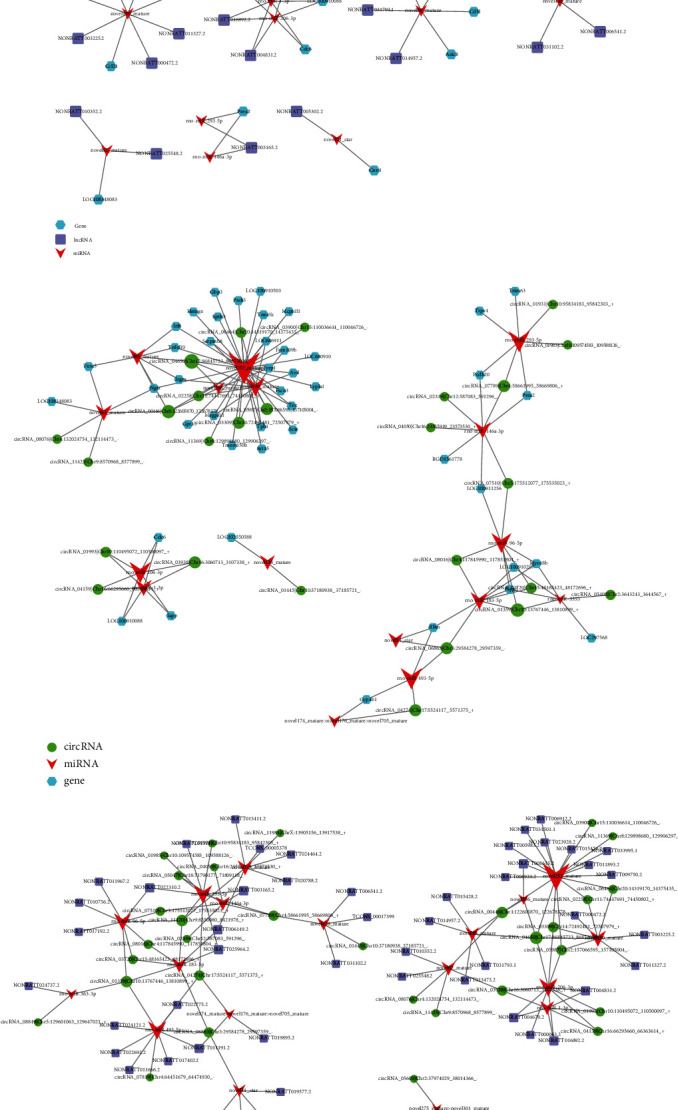
Construction of the mRNA-miRNA-lncRNA/circRNA network. (a) Network analysis of mRNA-miRNA-lncRNA. The blue nodes represent mRNA. The purple nodes represent lncRNA. The red nodes represent miRNA. (b) Network analysis of mRNA-miRNA-circRNA. The green nodes represent circRNA. The red nodes represent miRNA. The blue nodes represent mRNA. (c) Network analysis of lncRNA-miRNA-circRNA. The green nodes represent circRNA. The red nodes represent miRNA. The purple nodes represent lncRNA.

As shown in Figure 7(a), the network involves 30 lncRNAs, 35 mRNAs, and 13 miRNAs. At the same time, a circRNA-miRNA-mRNA ceRNA network was constructed (Figure 7(b)), involving 26 circRNAs, 44 mRNAs, and 16 miRNAs. Each differentially expressed lncRNA can be associated with one or more miRNAs. For example, lncRNA NONRATT016892.2 has established connections with two miRNAs, including rno-miR-1-3p and rno-miR-206-3p. miR-1187 is connected with four circRNAs, including circRNA_02339/Chr12:587083_591296, circRNA_07789/Chr4:58661995_58669806, circRNA_04050/Chr16:23555919_23573530, and circRNA_07510/Chr3:175512077_175535023. Finally, circRNA_01445/Chr10:37180938_37185721 has only established a connection with miR-438 (Figure 7(b)).

The two networks have multiple common nodes (Figure 7(c)), such as lncRNA NONRATT024121.2, lncRNA NONRATT022775.2, lncRNA NONRATT022692.2, lncRNA NONRATT011191.2, and lncRNA NONRATT017402.2, which all interact with miR-493-5p.

### 3.9. PPI Network and Functional Analysis of the Differentially Expressed mRNAs

**Figure 8:**
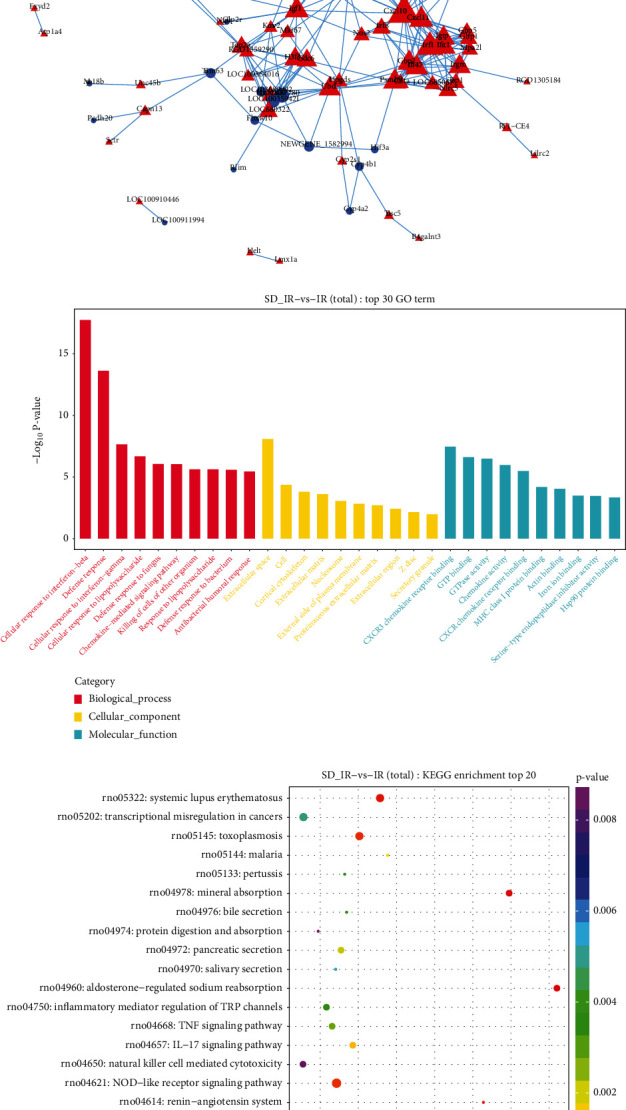
PPI network and functional analysis of the differentially expressed mRNAs. (a) The PPI network was constructed by differentially expressed mRNAs. (b) The top 30 Gene Ontology terms of differentially expressed mRNAs. (c) The top 20 KEGG pathway enrichment analysis of differentially expressed mRNAs.

To further address the most significant clusters of differentially expressed mRNAs in the ceRNA network, we conducted the PPI network analysis by using the STRING database version 11.0 and visualization under the Cytohubba plug-in and the Cytoscape. The most significant hub upregulated genes in the PPI network were Cxcl10, Cxcl11, Mmp9, Gbp2, Gbp5, Irgm, Mpa21, and Igf1, while the most significant hub downregulated genes were Ahsg, Trim63, and Trpv4 (Figure 8(a)).

To clarify the role of differential genes in the preventive effect of cardiac sympathetic denervation on MIRI, we performed GO and KEGG analyses on the differentially expressed mRNAs. The results suggested that the molecular functions (MF) are mainly enriched in the CXCR3 chemokine receptor binding, MHC class I protein binding, GTP binding, GTPase activity, and chemokine activity (Figure 8(b)). In the cell components (CC), functions are highly enriched in autophagy-related processes, which are related to the cortical cytoskeleton, nucleosomes, secretory granules. KEGG analysis showed that the differentially expressed mRNAs were involved in cytokine–cytokine receptor interaction, NOD-like receptor signaling pathway, chemokine signaling pathway, and inflammatory mediator regulation of TRP channels (Figure 8(c)). These results showed that most of the hub genes play a role in the preventive effect of cardiac sympathetic denervation on MIRI.

## 4. Discussion

This study provides novel information on the vital role of cardiac sympathetic denervation in the process of myocardial ischemia/reperfusion injury. Our main findings are as follows: (1) Cardiac sympathetic denervation induced by 6-OHDA alleviated myocardial ischemia/reperfusion injury. (2) The expression profiles of lncRNA, circRNA, and mRNA in the upper thoracic spinal cord were identified by RNA-seq analysis. Among them, there were 148 upregulated and 51 downregulated mRNAs, 165 upregulated and 168 downregulated lncRNAs, and 70 upregulated and 52 downregulated circRNAs in the SD-IR group compared with the IR group. (3) We selected three mRNAs from the most upregulated mRNAs and three lncRNAs from the most downregulated lncRNAs for RT-qPCR low-throughput verification, and the results were consistent with the sequencing results. By providing new insights into the function of lncRNA/circRNA-miRNA-mRNA networks, our results contribute to the understanding of the pathogenesis of MIRI and provide new targets for MIRI.

In recent years, a large number of studies have confirmed that cardiac sympathetic activity plays an important role in many cardiac diseases and processes [35–40]. Lu et al. reported that sympathetic hyperinnervation and/or myocardial infarction remodeled myocardial glutamate signaling and ultimately increased the severity of ventricular tachyarrhythmias [9]. It has also been shown that left stellate ganglion (LSG) suppression protects against ventricular arrhythmias. Yu et al. found that optogenetic modulation could reversibly inhibit the neural activity of LSG, thereby increasing electrophysiological stability and protecting against myocardial ischemia-induced ventricular arrhythmias [41]. These reports and our results also suggest that the presence of decreased cardiac sympathetic activity can have a cardioprotective effect, and that this depends on effective sympathetic denervation.

Recently, significant efforts have been made to understand the alterations of ncRNAs in different spinal cord segments and their contributions to specific outcomes of diseases [16, 26, 42–44]. The spinal cord is a complex and dynamic neural structure. It contains sympathetic preganglionic neurons within the intermediolateral cell column [45–47]; they are involved in the generation of sympathetic activity in many autonomic targets, including the heart and blood vessels [36, 48–50]. There is accumulating evidence of the interaction between the spinal cord and the heart [51–55]. We previously demonstrated the changes of novel lncRNAs in the upper thoracic spinal cord of rats with MIRI [42]. In recent years, there has been considerable effort to explore the relationship between cardiac sympathetic activity and cardiovascular diseases. However, the changes in spinal lncRNAs in rats with MIRI after cardiac sympathetic denervation have not been reported. Here, we aimed to understand the involvement of specific patterns of changes in the lncRNA/circRNAs-miRNA-mRNA network of the upper thoracic spinal cord regions of animals with myocardial ischemia-reperfusion injury after cardiac sympathetic denervation.

LncRNAs are involved in the progression of coronary artery disease (CAD) [56]. Xu et al. reported that lncRNA AC096664.3/PPAR-gamma/ABCG1-dependent signal transduction pathway contributes to the regulation of cholesterol homeostasis [56]. As one of the differentially expressed lncRNAs between CAD patients and healthy controls, lncRNA ENST00000602558.1 plays a key role in the pathogenesis of atherosclerosis. Cai et al. showed that lncRNA ENST00000602558.1 regulated ABCG1 expression and cholesterol efflux from vascular smooth muscle cells through a p65-dependent pathway [57]. The study by Li et al. provided the characterization of lncRNA expression profile and identification of novel lncRNA biomarkers to diagnose CAD [58]. According to our study, spinal lncRNA as a sponge of miRNA mainly participates in the process of MIRI through cysteine and methionine metabolism, mTOR signaling pathway, insulin signaling pathway, and adipocytokine signaling pathway.

Circular RNAs (circRNAs) play a critical role in the physiology and pathology of cardiovascular diseases [59–62]. To further investigate the roles of these differentially expressed circRNAs in the development of MIRI, we performed GO and KEGG pathway analyses. Based on the GO and KEGG enrichment analyses of these circRNAs, our results suggested that the significantly enriched biologic processes and molecular functions of the upregulated genes after MIRI were associated with gene sets termed as follows: “MAPK signaling pathway” and “cGMP-PKG signaling pathway”. It is well known that MAPK pathway is involved in ischemia-reperfusion injury [63]. Previous studies have shown that cGMP-PKG pathways are implicated in cardiovascular complications of diverse etiologies [64, 65]. These data suggest that spinal circRNAs may be potential targets for MIRI.

miRNAs have been shown to modulate the translational activity of the genome and regulate protein expression and function [66–68]. According to Wang et al. [69], miRNA-493-5p promotes apoptosis and suppresses proliferation and invasion in liver cancer cells by targeting VAMP2. Previous studies have pointed out a potential cardioprotective role of phosphatidylserine in heart ischemia [70–73], suggesting that the phosphatidylserine signaling pathway is associated with MIRI. Schumacher et al. [74] indicated that phosphatidylserine significantly reduced the infarct size by 30% and improved heart function by 25% in a chronic model of acute myocardial infarction (AMI), suggesting that phosphatidylserine supplementation may be a promising novel strategy to reduce infarct size following AMI and to prevent myocardial injury during myocardial infarction or cardiac surgery. A large number of studies have confirmed that chemokines [75–77], including C-X-C motif chemokine receptor 3 (CXCR3) [78], are closely related to the ischemia–reperfusion injury. In this study, we found that miRNAs in the spinal cord participated in the molecular progression of MIRI through the regulation of actin cytoskeleton, phospholipase D, calcium, and MAPK signaling pathways.

It has been found that the lncRNA/circRNA-miRNA-mRNA ceRNA network plays a role in multiple physiological and pathological processes [66, 79–86]. Cheng et al. [82] reported the comprehensive analysis of the circRNA-lncRNA-miRNA-mRNA ceRNA network in the prognosis of acute myeloid leukemia (AML), elucidated the post-transcriptional regulatory mechanism of AML, and identified novel AML prognostic biomarkers, which has important guiding significance for the clinical diagnosis, treatment, and further scientific research of AML. Wang et al. [87] established bronchopulmonary dysplasia (BPD)-related ceRNA regulatory network of circRNA/lncRNA-miRNA-mRNA in the lung tissue of a mouse model, proving that it is significantly associated with the pathophysiological characteristics of BPD. In this study, we analyzed the changes in spinal lncRNA-miRNA-mRNA and circRNA-miRNA-mRNA ceRNA networks in MIRI after cardiac sympathetic denervation. Our findings offer a new direction for understanding the pathogenesis of MIRI, and suggest some effective targets in the spinal cord after cardiac sympathetic denervation.

In conclusion, the expression characteristics of coding genes, miRNAs, lncRNAs, and circRNAs in the upper thoracic spinal cord of MIRI rats were determined after cardiac sympathetic denervation induced by 6-OHDA. The preventive effect of cardiac sympathetic denervation on MIRI paves the road for further studies on the sympathetic mechanisms associated with MIRI, which is important to further explore the pathogenesis of MIRI and potentially facilitate the discovery of novel lncRNA/circRNA-miRNA-mRNA networks for therapeutic targeting in the management of MIRI.

## Figures and Tables

**Table 1:** Primer sequences for reverse transcriptase-quantitative polymerase chain reaction.

Primer name	Primer sequences (5′ to 3′)
*LncRNA*	
NONRATT012797.2-F	CTGGGGTGAGAAGGGCTGAC
NONRATT012797.2-R	AAGGTGTTTTCCCGGAGGGC
NONRATT029190.2-F	ACTGGGGTGGCACTTAGAGG
NONRATT029190.2-R	TGTCCACCGTAACATCCCCT
NONRATT000247.2-F	CAGGGCCTTGTGCTTGCTA
NONRATT000247.2-R	ATGTTTTCCCTCCGCTGCTT
NONRATT004098.2-F	ACCTCTTCCCCTCAGCCTACAG
NONRATT004098.2-R	TCACTGCCATGAATCACATTCCA
NONRATT025664.2-F	ATGCCAACCTTACTATACGTTTCC
NONRATT025664.2-R	TGACTCTCCCACCAACTTCAG
*mRNA*	
Ubd-F	GGTGAAGCCCAGTGATGAAGAGC
Ubd-R	GGGAGGCACAGCAGTCACATTC
Ccl12-F	CTGCTCATAGCTGCCGCCATC
Ccl12-R	GCCTCCGAATGTGGATCTTCTGC
Cxcl10-F	GTTCTCTGCCTCGTGCTGCTG
Cxcl10-R	AACATGCGGACAGGATAGACTTGC
LOC100912599-F	GCAGTTCAAGCAGCAGCATCAC
LOC100912599-R	AACAAGGGACACCATTCACAGAGC
Dpep1-F	CATCGCATGTGCCAGCTCTATCC
Dpep1-R	GCCACCTTCCACGCCAATCAG
GAPDH-F	GACATGCCGCCTGGAGAAAC
GAPDH-R	AGCCCAGGATGCCCTTTAGT

**Table 2:** The detail information of the top 30 up-regulated mRNAs in the T1-4 spinal cord between SD-IR group and IR group.

Gene ID	log_2_FC (SD-IR/IR)	Pvalue	Description
LOC102553010	4.40	0.021887	Leukocyte elastase inhibitor A-like
RGD1359290	4.10	0.019123	Ribosomal_L22 domain containing protein RGD1359290
LOC108348083	3.91	0.014045	Delta-1-pyrroline-5-carboxylate synthase
RGD1305184	3.83	0.011827	Similar to CDNA sequence BC023105
Gns	3.81	0.012511	Glucosamine (N-acetyl)-6-sulfatase
Prf1	3.57	0.003838	Perforin 1
LOC100911034	3.47	0.020641	Cysteine desulfurase, mitochondrial-like
Ubd	2.99	0.024065	Ubiquitin D
Mcpt9	2.96	0.016414	Mast cell protease 9
LOC100910446	2.95	0.039393	Syntaxin-7-like
Sctr	2.94	0.009767	Secretin receptor
Ccl12	2.92	0.000115	Chemokine (C-C motif) ligand 12
Cxcl10	2.89	0.011969	C-X-C motif chemokine ligand 10
Ngp	2.81	0.000296	Neutrophilic granule protein
Rhag	2.78	0.001015	Rh-associated glycoprotein
Cxcl11	2.74	0.020485	C-X-C motif chemokine ligand 11
Car1	2.64	2.06E-05	Carbonic anhydrase I
Padi3	2.50	0.02082	Peptidyl arginine deiminase 3
S100a5	2.43	0.031729	S100 calcium binding protein A5
Unc45b	2.19	0.016491	Unc-45 myosin chaperone B
Cd5l	2.10	0.034827	Cd5 molecule-like
Ermap	2.03	0.010957	Erythroblast membrane-associated protein
Capn13	1.94	0.046362	Calpain 13
Clec5a	1.87	3.08E-05	C-type lectin domain family 5, member A
Gbp1	1.84	0.040079	Guanylate binding protein 1
Hemgn	1.77	8.40E-05	Hemogen
Cxcl13	1.76	0.00143	C-X-C motif chemokine ligand 13
Kel	1.76	0.00334	Kell blood group, metallo-endopeptidase
LOC680322	1.75	0.028182	Similar to histone H2A type 1
Ms4a3	1.75	0.003821	Membrane spanning 4-domains A3

|log2FC| > 1; p < 0.05 by analysis of variance.

**Table 3:** The detail information of the top 30 down-regulated mRNAs in the T1-4 spinal cord between SD-IR group and IR group.

Gene ID	log_2_FC (SD-IR/IR)	Pvalue	Description
LOC100912599	-5.31	0.010395	NADH dehydrogenase [ubiquinone] iron-sulfur protein 6, mitochondrial-like
LOC100911994	-3.89	0.01095	Coiled-coil domain-containing protein 132-like
Clcn2	-3.51	0.029527	Chloride channel, voltage-sensitive 2
LOC100910308	-3.47	7.97E-05	Multifunctional protein ADE2-like
LOC100910207	-3.12	0.018014	Protein Dr1-like
Vwa5a	-2.62	0.02532	von Willebrand factor A domain containing 5A
Dpep1	-2.53	0.015698	Dipeptidase 1 (renal)
NEWGENE_1582994	-2.45	0.02675	DCN1, defective in cullin neddylation 1, domain containing 2
LOC103689986	-2.41	1.64E-06	Protein YIF1B
Iqcf3	-2.40	0.036362	IQ motif containing F3
Ahsg	-2.32	0.021173	Alpha-2-HS-glycoprotein
Hpgd	-2.01	0.008083	Hydroxyprostaglandin dehydrogenase 15 (NAD)
RGD1561778	-1.94	0.016898	Similar to dendritic cell-derived immunoglobulin(Ig)-like receptor 1, DIgR1 - mouse
LOC297568	-1.88	0.032025	Alpha-1-inhibitor III
Rlim	-1.78	0.042544	Ring finger protein, LIM domain interacting
Slc39a12	-1.65	0.017633	Solute carrier family 39 member 12
LOC100911865	-1.62	1.20E-02	TBC1 domain family member 12-like
Myo18b	-1.61	0.003815	Myosin XVIIIb
Tspan10	-1.59	0.012324	Tetraspanin 10
Sds	-1.57	0.025502	Serine dehydratase
Trim63	-1.39	0.010581	Tripartite motif containing 63
LOC102550588	-1.38	0.030436	Zinc finger protein 709-like
Sh2d4b	-1.37	2.44E-05	SH2 domain containing 4B
Hif3a	-1.35	0.002856	Hypoxia inducible factor 3, alpha subunit
Galnt15	-1.30	0.01374	Polypeptide N-acetylgalactosaminyltransferase 15
LOC687780	-1.30	0.029663	Similar to Finkel-Biskis-Reilly murine sarcoma virusubiquitously expressed
Trpv4	-1.29	0.015774	Transient receptor potential cation channel, subfamily V, member 4
Mt1	-1.18	0.033903	Metallothionein 1
Fbxw10	-1.11	0.016073	F-box and WD repeat domain containing 10
Dcst1	-1.10	0.024549	DC-STAMP domain containing 1

|log2FC| > 1; p < 0.05 by analysis of variance.

**Table 4:** The detail information of the top 30 up-regulated lncRNAs and top 30 down-regulated lncRNAs in the T1-4 spinal cord between SD-IR group and IR group.

Upregulation			Downregulation		
LncRNA ID	log_2_FC (SD-IR/IR)	Pvalue	LncRNA ID	log_2_FC (SD-IR/IR)	Pvalue
NONRATT015643.2	6.61	0.030213	NONRATT002082.2	-8.80	0.003863
NONRATT003628.2	6.28	0.006736	NONRATT004821.2	-8.62	0.025764
NONRATT005973.2	6.21	0.009866	NONRATT010722.2	-7.18	0.000854
NONRATT016279.2	6.00	0.004952	NONRATT000247.2	-5.80	0.00261
NONRATT008379.2	5.61	0.014618	NONRATT027814.2	-5.77	0.012322
NONRATT013717.2	5.58	0.036523	NONRATT008629.2	-5.75	0.017423
NONRATT016674.2	5.56	0.047236	NONRATT005321.2	-5.65	0.002079
NONRATT017458.2	5.44	0.008762	NONRATT028463.2	-5.59	0.000887
NONRATT002859.2	5.27	0.016794	TCONS_00021543	-5.51	0.006752
NONRATT009760.2	5.22	0.028265	NONRATT004098.2	-5.28	0.011345
TCONS_00002734	5.09	0.011358	NONRATT007713.2	-5.20	3.10E-05
NONRATT011768.2	4.95	0.01038	NONRATT004090.2	-5.07	0.010222
NONRATT009811.2	4.73	0.000946	NONRATT018759.2	-5.06	0.001982
NONRATT026200.2	4.54	0.004819	NONRATT003447.2	-5.03	0.010344
NONRATT030638.2	4.44	0.025926	NONRATT007815.2	-5.02	0.039319
NONRATT008615.2	4.37	0.001033	NONRATT025664.2	-4.82	5.13E-05
NONRATT012797.2	4.35	0.023183	NONRATT019695.2	-4.69	0.008267
NONRATT020189.2	4.33	0.011361	NONRATT006541.2	-4.66	3.19E-11
NONRATT020809.2	4.27	0.02875	NONRATT019538.2	-4.60	0.013069
NONRATT029190.2	4.25	0.001325	NONRATT026641.2	-4.54	0.01747
NONRATT022867.2	4.24	0.031209	NONRATT024737.2	-4.38	0.010641
NONRATT000517.2	4.24	0.031431	TCONS_00013588	-4.30	6.80E-06
NONRATT003681.2	4.18	0.012182	NONRATT020675.2	-4.21	0.010623
NONRATT021791.2	4.16	0.009107	NONRATT010709.2	-4.20	0.043975
NONRATT023302.2	4.14	0.038514	NONRATT022516.2	-4.14	0.048093
NONRATT003838.2	4.12	0.043331	NONRATT020315.2	-4.13	0.01375
NONRATT018320.2	4.10	0.005454	TCONS_00002731	-4.02	0.028687
NONRATT013257.2	3.87	0.043965	NONRATT006149.2	-3.89	0.003669
NONRATT000472.2	3.79	0.048619	NONRATT011296.2	-3.87	0.033214
NONRATT005188.2	3.76	0.019322	TCONS_00007997	-3.75	0.001282

|log2FC| > 1; p < 0.05 by analysis of variance.

**Table 5:** The detail information of the top 20 up-regulated circRNAs in the T1-4 spinal cord between SD-IR group and IR group.

CircRNA ID	log_2_FC (SD-IR/IR)	Pvalue
circRNA_06761|Chr3:13146571_13147147_-	3.91	0.000446
circRNA_03748|Chr15:51936366_51954838_-	3.80	0.000648
circRNA_01962|Chr10:103586432_103677527_-	3.27	0.017323
circRNA_11924|Chr9:119105074_119127222_+	3.16	0.019308
circRNA_01988|Chr10:109580083_109583063_-	3.16	0.01944
circRNA_04213|Chr16:81879800_81897683_-	3.10	0.027871
circRNA_05436|Chr2:5570780_5576549_-	3.08	0.047573
circRNA_09388|Chr6:38617368_38619590_+	3.06	0.03965
circRNA_04650|Chr17:86845723_86873000_-	2.89	0.013496
circRNA_02217|Chr11:70539679_70570814_-	2.74	0.026695
circRNA_06237|Chr2:218919131_218929338_-	2.69	0.040249
circRNA_11356|Chr8:128384378_128391077_-	2.61	0.04823
circRNA_08473|Chr5:50318351_50319000_-	2.60	0.036427
circRNA_02159|Chr11:47165426_47169764_+	2.56	0.001643
circRNA_05629|Chr2:45801786_45859187_+	2.41	0.033552
circRNA_09485|Chr6:60609626_60633969_+	2.26	0.009238
circRNA_06869|Chr3:33465220_33473531_+	2.22	0.023101
circRNA_00547|Chr1:143712491_143723739_-	2.19	0.006884
circRNA_08772|Chr5:123824724_123825786_+	2.15	0.005133
circRNA_01552|Chr10:55283300_55338553_+	2.06	0.028481

|log2FC| > 1; p < 0.05 by analysis of variance.

**Table 6:** The detail information of the top 20 down-regulated circRNAs in the T1-4 spinal cord between SD-IR group and IR group.

CircRNA ID	log_2_FC (SD-IR/IR)	Pvalue
circRNA_06863|Chr3:29584278_29597359_-	-3.41	0.004976
circRNA_11934|Chr9:119750465_119765191_-	-3.34	0.007029
circRNA_02911|Chr13:92762599_92783433_+	-3.28	0.009623
circRNA_04274|Chr17:5524117_5571375_+	-3.25	0.013595
circRNA_04457|Chr17:53489461_53499989_+	-3.20	0.013695
circRNA_11984|ChrX:13905156_13917538_+	-2.97	0.035854
circRNA_08837|Chr5:129413066_129491380_+	-2.89	0.048702
circRNA_03573|Chr15:13454469_13468631_-	-2.26	0.019466
circRNA_07224|Chr3:113195876_113197193_-	-2.26	0.016518
circRNA_07819|Chr4:64451679_64474930_-	-2.24	0.021549
circRNA_04050|Chr16:23555919_23573530_+	-2.12	0.00851
circRNA_09970|Chr7:9826042_9826424_-	-1.97	0.035185
circRNA_11137|Chr8:96067918_96073130_-	-1.61	0.040373
circRNA_07862|Chr4:66193731_66214585_+	-1.57	0.049364
circRNA_07510|Chr3:175512077_175535023_+	-1.57	0.038954
circRNA_11420|Chr9:8350080_8421978_+	-1.53	0.007153
circRNA_03499|Chr14:113838631_113857380_+	-1.40	0.04037
circRNA_09492|Chr6:64852693_64861750_+	-1.32	0.035057
circRNA_02339|Chr12:587083_591296_-	-1.19	0.014725
circRNA_04622|Chr17:84855705_84901793_+	-1.03	0.038253

|log2FC| > 1; p < 0.05 by analysis of variance.

**Table 7:** The detail information of differentially expressed miRNAs in the T1-4 spinal cord between SD-IR group and IR group.

miRNA_ID	log_2_FC (SD-IR/IR)	Pvalue	Length
Upregulation			
novel21_star	3.695484	0.021677	23
Rno-miR-293-5p	2.798924	0.000538	21
novel98_mature	2.479701	0.03268	23
novel174_mature>novel176_mature>novel705_mature	2.236875	0.046924	22
Rno-miR-146a-3p	2.118125	0.026213	21
Rno-miR-96-5p	0.903083	0.005817	23
Rno-miR-183-5p	0.614791	0.003377	22
Rno-miR-493-5p	0.60771	0.007994	22
Rno-miR-363-3p	0.60157	0.019368	21
Rno-miR-3553	0.600458	0.03527	23
Downregulation			
novel190_mature	-3.63316	0.016898	23
novel275_mature>novel301_mature	-3.4838	0.045199	24
novel586_mature	-3.41968	0.000414	23
novel88_mature	-2.24793	0.005616	23
novel252_mature	-1.81632	0.044256	23
novel62_mature	-1.46827	0.03968	24
Rno-miR-206-3p	-1.07274	0.014845	22
Rno-miR-1-3p	-0.86313	0.029666	22
Rno-miR-7a-2-3p	-0.67697	0.040237	22

|log2FC| > 1; p < 0.05 by analysis of variance.

**Table 8:** The Gene Ontology (GO) terms enriched for the differentially expressed genes.

GO ID	Term	Gene number	Pvalue
biological_process			
GO:0035458	Cellular response to interferon-beta	12	1.80E-18
GO:0006952	Defense response	11	2.39E-14
GO:0071346	Cellular response to interferon-gamma	9	2.22E-08
GO:0071222	Cellular response to lipopolysaccharide	11	2.09E-07
GO:0050832	Defense response to fungus	4	8.84E-07
GO:0070098	Chemokine-mediated signaling pathway	6	9.17E-07
GO:0031640	Killing of cells of other organism	4	2.35E-06
GO:0032496	Response to lipopolysaccharide	12	2.41E-06
GO:0042742	Defense response to bacterium	8	2.62E-06
GO:0019731	Antibacterial humoral response	4	3.59E-06
cellular_component			
GO:0005615	Extracellular space	34	8.43E-09
GO:0005623	Cell	6	4.24E-05
GO:0030863	Cortical cytoskeleton	3	0.00016
GO:0031012	Extracellular matrix	8	0.000245
GO:0000786	Nucleosome	4	0.000876
GO:0009897	External side of plasma membrane	8	0.001475
GO:0005578	Proteinaceous extracellular matrix	7	0.001944
GO:0005576	Extracellular region	13	0.003785
GO:0030018	Z disc	4	0.006991
GO:0030141	Secretory granule	4	0.010644
molecular_function			
GO:0048248	CXCR3 chemokine receptor binding	3	3.51E-08
GO:0005525	GTP binding	15	2.48E-07
GO:0003924	GTPase activity	13	3.24E-07
GO:0008009	Chemokine activity	5	1.05E-06
GO:0045236	CXCR chemokine receptor binding	3	3.30E-06
GO:0042288	MHC class I protein binding	3	6.49E-05
GO:0003779	Actin binding	9	9.09E-05
GO:0005506	Iron ion binding	7	0.00032
GO:0004867	Serine-type endopeptidase inhibitor activity	5	0.000333
GO:0051879	Hsp90 protein binding	3	0.000451

**Table 9:** The Kyoto Encyclopedia of Genes and Genomes (KEGG) pathways enriched for the differentially expressed genes.

Pathway ID	Term	Gene number	Pvalue
rno04960	Aldosterone-regulated sodium reabsorption	4	2.24E-05
rno04978	Mineral absorption	4	4.64E-05
rno05322	Systemic lupus erythematosus	5	0.000233
rno04614	Renin-angiotensin system	3	0.000307
rno04060	Cytokine-cytokine receptor interaction	8	0.00035
rno05145	Toxoplasmosis	5	0.000464
rno04621	NOD-like receptor signaling pathway	6	0.000495
rno04657	IL-17 signaling pathway	4	0.001424
rno05144	Malaria	3	0.00156
rno04972	Pancreatic secretion	4	0.00207
rno04668	TNF signaling pathway	4	0.002791
rno04750	Inflammatory mediator regulation of TRP channels	4	0.003408
rno04976	Bile secretion	3	0.003981
rno05133	Pertussis	3	0.004186
rno05202	Transcriptional misregulation in cancers	5	0.004801
rno04062	Chemokine signaling pathway	5	0.004938
rno04970	Salivary secretion	3	0.00532
rno00830	Retinol metabolism	3	0.006364
rno04650	Natural killer cell mediated cytotoxicity	4	0.00852
rno04974	Protein digestion and absorption	3	0.008842

## Data Availability

The raw sequencing data presented in this paper have been deposited in the Sequence Read Archive (SRA) under accession number PRJNA776390. The records are accessible with the following link: https://www.ncbi.nlm.nih.gov/sra/PRJNA776390.
